# Identification of the First Single *GSDME* Exon 8 Structural Variants Associated with Autosomal Dominant Hearing Loss

**DOI:** 10.3390/diagnostics12010207

**Published:** 2022-01-15

**Authors:** Luke Mansard, Christel Vaché, Julie Bianchi, Corinne Baudoin, Isabelle Perthus, Bertrand Isidor, Catherine Blanchet, David Baux, Michel Koenig, Vasiliki Kalatzis, Anne-Françoise Roux

**Affiliations:** 1 Molecular Genetics Laboratory, Univ Montpellier, CHU Montpellier, 34000 Montpellier, France; l-mansard@chu-montpellier.fr (L.M.); j-bianchi@chu-montpellier.fr (J.B.); corinne.baudoin@inserm.fr (C.B.); david.baux@inserm.fr (D.B.); michel.koenig@inserm.fr (M.K.); anne-francoise.roux@inserm.fr (A.-F.R.); 2 Institute for Neurosciences of Montpellier (INM), Univ Montpellier, Inserm, 34000 Montpellier, France; vasiliki.kalatzis@inserm.fr; 3 Department of Clinical Genetics, Reference Center for Rare Diseases, University Hospital of Clermont-Ferrand, 63000 Clermont-Ferrand, France; iperthus@chu-clermontferrand.fr; 4 Department of Medical Genetics, CHU Nantes, 44000 Nantes, France; Bertrand.ISIDOR@chu-nantes.fr; 5 National Reference Centre for Inherited Sensory Diseases, Otolaryngology Department, Univ Montpellier, CHU Montpellier, 34000 Montpellier, France; c-blanchet@chu-montpellier.fr

**Keywords:** *GSDME*, *DFNA5*, hearing loss, single-exon CNV

## Abstract

*GSDME*, also known as *DFNA5*, is a gene implicated in autosomal dominant nonsyndromic hearing loss (ADNSHL), affecting, at first, the high frequencies with a subsequent progression over all frequencies. To date, all the *GSDME* pathogenic variants associated with deafness lead to skipping of exon 8. In two families with apparent ADNSHL, massively parallel sequencing (MPS) integrating a coverage-based method for detection of copy number variations (CNVs) was applied, and it identified the first two causal *GSDME* structural variants affecting exon 8. The deleterious impact of the c.991-60_1095del variant, which includes the acceptor splice site sequence of exon 8, was confirmed by the study of the proband’s transcripts. The second mutational event is a complex rearrangement that deletes almost all of the exon 8 sequence. This study increases the mutational spectrum of the *GSDME* gene and highlights the crucial importance of MPS data for the detection of *GSDME* exon 8 deletions, even though the identification of a causal single-exon CNV by MPS analysis is still challenging.

## 1. Introduction

The Gasdermin E gene (*GSDME*), also called deafness autosomal dominant 5 (*DFNA5*), located on chromosome 7p15, contains 10 exons and encodes the 496-amino acid Gasdermin-E protein. This protein, which is a member of the Gasdermin superfamily, displays a necrotic-inducing N-terminal domain (GSDME-N, amino acids 1 to 270) self-inhibited by a C-terminal domain (GSDME-C, amino acids 271 to 496) [1]. When the connection between these two domains is cleaved by the apoptotic protease caspase-3 or the killer cell granzyme B (GzmB), the released GSDME-N chain participates in the cell death pathway by forming pores in the plasma and mitochondrial membranes [2,3].

**Figure A1:**
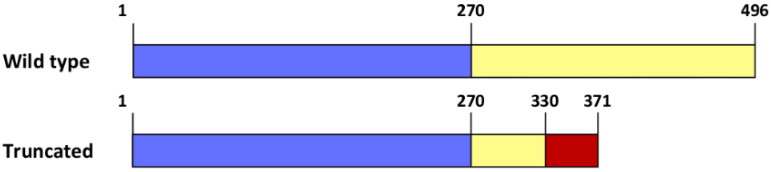
Schematic representation of the wild-type and truncated Gasdermin-E proteins. The necrotic N-terminal domain is represented in blue, and the C-terminal domain in yellow. The aberrant 41-amino acid tail of the truncated protein is represented in red.

*GSDME* is considered as a potential tumor suppressor gene (for review, see [4]), and downregulation or suppression of its necrotic function has been observed in several cancers [3]. Gain-of-function pathogenic variants in *GSDME* have also been reported, but they all lead to exon 8 skipping at the mRNA level and result in autosomal, dominant, progressive, sensorineural and nonsyndromic DFNA5 hearing loss (OMIM #600994). These variations are located in the flanking sequences of exon 8 or within the exon itself. They alter the splice consensus sequences [5,6,7,8,9,10,11,12], impact the polypyrimidine tract [13] or disturb regulatory elements [10,14]. This out-of-frame exon 8 skipping results in the production of a C-terminally truncated, constitutively active necrotic protein (Figure A1).

In this article, we describe the molecular analysis of two unrelated families suffering from progressive nonsyndromic hearing loss with an apparent dominant inheritance, and the identification of two copy number variations (CNVs) affecting the single *GSDME* exon 8 as the causal variants.

## 2. Materials and Methods

### 2.1. Clinical Report of the Families

#### 2.1.1. Family S2426

**Figure 1:**
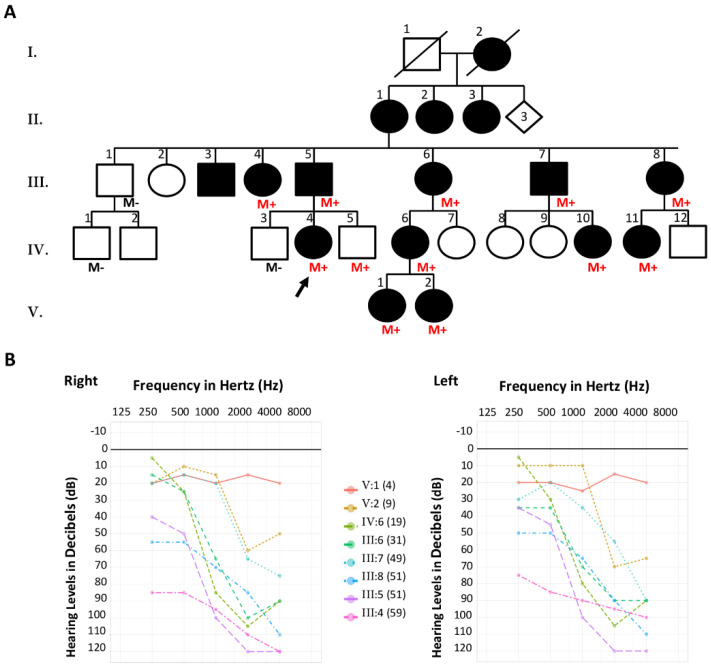
Family S2426. (**A**) Pedigree of the family. Filled symbols denote affected individuals. The proband referred for molecular testing is indicated by a black arrow. M+ (red font): presence of the *GSDME* deletion, M− (black font): absence of the *GSDME* deletion. (**B**) Superimposed pure-tone audiograms of eight affected individuals. The numbers in parentheses indicate the subject’s age at the audiometric testing. Left chart: right ear, right chart: left ear.

Family S2426 was a large French family with five generations, including sixteen members affected by nonsyndromic hearing loss (NSHL) with a dominant pattern of inheritance (Figure 1A). Family history reported post-lingual bilateral progressive hearing loss with an assumed onset in the first or second decade of life in all affected individuals. Superimposed audiograms (pure-tone audiometry at 250, 500, 1000, 2000 and 4000 Hz) from eight of the affected members (III:4, III:5, III:6, III:7, III:8, IV:6, V:1 and V:2) performed at different ages (59, 51, 31, 49, 51, 19, 4 and 9 years, respectively) confirmed progressive moderate to profound hearing loss that initially affected the high frequencies (downward-sloping curve) and then progressed across all frequencies (Figure 1B). The proband referred for molecular testing was a 55-year-old woman (IV:4) suffering from progressive hearing loss detected when she was 6 years old. Clinical examination of all available affected individuals of this family was otherwise unremarkable.

#### 2.1.2. Family S2106

**Figure 2:**
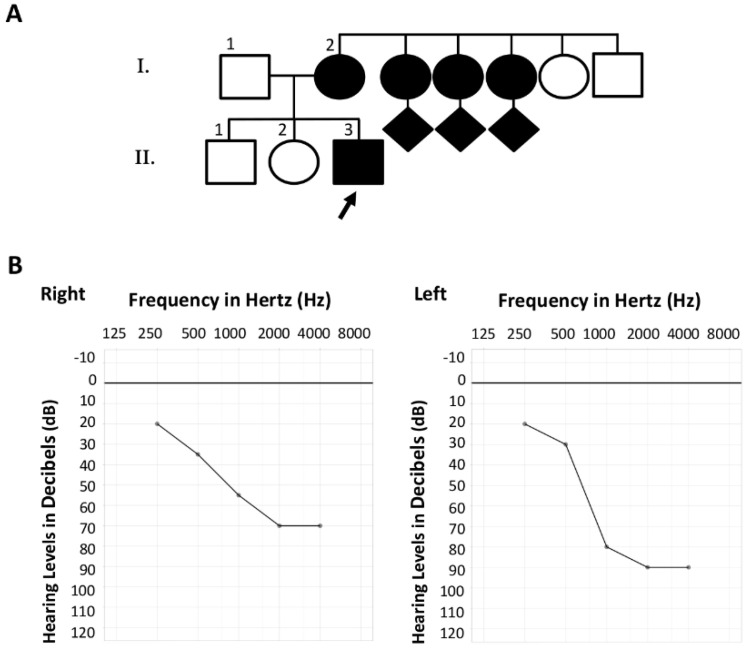
Family S2106. (**A**) Pedigree of the family. Filled symbols denote affected individuals. The proband referred for molecular testing is indicated by a black arrow. (**B**) Pure-tone audiogram of the patient at the age of 30 years old. Left chart: right ear, right chart: left ear.

Based on information obtained from the proband II:3, his family was composed of at least two generations including more than ten members presenting with NSHL. A likely autosomal dominant inheritance pattern of the disease was suspected (Figure 2A), although no precise clinical data on other affected members could be obtained. The hearing impairment of the proband (II:3) was diagnosed when he was 6 years old, and physical examination did not find any evidence of a syndromic disease. His deafness was progressive, and available data from an audiometric assessment at the age of 30 years old (Figure 2B) identified bilateral asymmetrical hearing loss with downward-sloping curves. These audiograms revealed pure-tone averages (PTAs) of 57.5 db HL and 72.5 db HL for the right and left ears, respectively. Due to substantial PTA differences across the right and left ears, and in accordance with the 02/1bis recommendation of the International Bureau for Audiophonology (BIAP; https://www.biap.org/ (accessed on 8 December 2021)), a PTA of 62 db HL was retained, corresponding to moderate group 2 hearing loss.

### 2.2. DNA Analysis

Genomic DNA from the 15 participating family members from family S2426 and the proband from family S2106 was isolated from peripheral blood samples using standard procedures. The DNA of the two probands was analyzed by massively parallel sequencing (MPS) using a hearing loss gene panel on an Illumina MiniSeq sequencer (Illumina, San Diego, CA, USA). The screened genes and the complete workflow used to identify pathogenic alterations have already been described [15]. This molecular diagnosis strategy included a copy number estimation of each region by a depth of coverage- based method using the MobiCNV algorithm (https://github.com/mobidic/MobiCNV (accessed on 23 November 2021)), which was completed by a direct visualization of the sequenced reads with the open source Integrative Genomics Viewer (IGV) software (v2.7.2) [16].

Validation and familial segregation (when possible) of the *GSDME* variations were conducted by PCR-Sanger sequencing using the BigDye Terminator v3.1 cycle sequencing kit (Applied Biosystems, Courtaboeuf, France) on an Applied Biosytems^®^ 3500Dx Genetic Analyzer (Applied Biosystems). PCRs were performed with the forward primer 5′-GAGGAATTTCCATCCATTTGC-3′ combined with the reverse primer 5′-CACAGTGTGGGAATGATCTGG-3′ for S2426, and the forward primer 5′-CCCGTCAGTGAAATGTAGCC-3′ paired with the reverse primer 5′-CTCTGTGTCCCCAGAAGCA-3′ for S2106.

### 2.3. RNA Analysis

The functional consequence of the *GSDME* variant identified in family S2426 was investigated by RNA analysis. Total RNA was isolated from whole blood collected in PAXgeneTM Blood RNA Tubes using the Nucleo Spin^®^ RNA II isolation kit (Macherey-Nagel, Düren, Germany). Reverse transcription was performed using the SuperScriptTM III Reverse Transcriptase (Invitrogen, Carlsbad, CA, USA) and oligo (dT) primers, according to the manufacturer’s instructions. PCRs were then carried out with *GSDME*-specific primers (forward: 5′-CACAGTGTGGGAATGATCTGG-3′, reverse: 5′-TTCAGGGGAGTCAAGGTTGG-3′), and amplicons were Sanger sequenced.

### 2.4. Variant Description

The nomenclature of the variants follows the Human Genome Variation Society (HGVS) recommendations v20.05 (http://varnomen.hgvs.org/ (accessed on 23 November 2021)) [17], with nucleotide +1 corresponding to the A of the ATG initiation codon in the *GSDME* reference sequence NM_004403.2; NG_011593.1. The two *GSDME* variants have been added to the Leiden Open Variation Database Global Variome Shared Instance (LOVD GVShared, https://databases.lovd.nl/shared/variants/DFNA5 (accessed on 17 December 2021)) and classified in accordance with the adapted ACMG/AMP guidelines for variant interpretation in the context of hearing loss [18].

Several DNA variation databases, including the Human Gene Mutation Database (HGMD^®^ Professional 2020.3; https://portal.biobase-international.com), the Genome Aggregation Database (gnomAD) (https://gnomad.broadinstitute.org/), the Single Nucleotide Polymorphism Database (dbSNP) (https://www.ncbi.nlm.nih.gov/snp/), the Clinical Variation Database (ClinVar) (https://www.ncbi.nlm.nih.gov/clinvar/), the Deafness Variation Database (DVD) (https://deafnessvariationdatabase.org/) and the LOVD GVShared, were accessed on 8 December 2021.

## 3. Results

### 3.1. Family S2426

**Figure 3:**
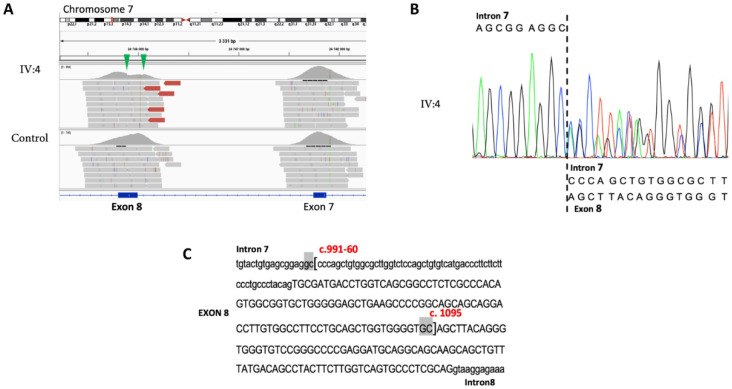
Identification and validation of the *GSDME* deletion in the proband IV:4. (**A**) Integrative genomics viewer screenshot focused on the *GSDME* exons 8 and 9. The sequence reads from the proband and a control are shown. Green arrowheads indicate the position of the deletion. (**B**) Sequence chromatogram of the c.991-60_1095del variant. (**C**) Sequence context of the deletion. The brackets indicate the 5′ and 3′ breakpoints of the deletion. The 2 bp flanking microhomologies are highlighted in gray.

Analyses of the MPS data obtained from proband IV:4, using the MobiCNV algorithm, pointed out a depth of coverage decrease in the *GSDME* gene compatible with a potential exon 8 deletion in the heterozygous state. Visualization of the sequenced reads with the IGV tool confirmed this CNV and identified the breakpoints of the deletion (Figure 3A). The presence of this c.991-60_1095del variant was validated by Sanger sequencing in the proband’s DNA (Figure 3B), and flanking microhomologies of 2 bp were observed (Figure 3C).

**Figure 4:**
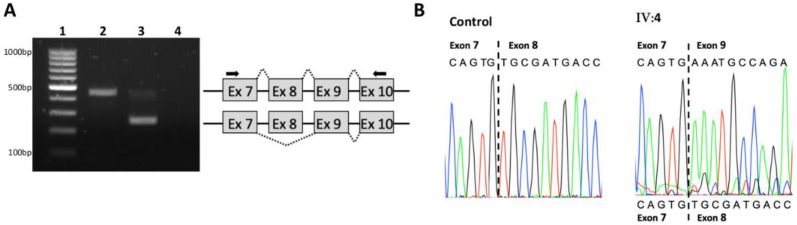
(**A**) RNA analysis of the c.991-60_1095del variant. (**A**) Agarose gel electrophoresis of the RT-PCR products from a control (lane 2) and the proband IV:4 (line 3). Lane 1: molecular weight markers, lane 4: control PCR reaction without template. A schematic representation of the spliced products is included. The position of the primers used for the amplification is shown by arrows. (**B**) Sanger sequencing electropherograms of the RT-PCR products for the control and the patient.

However, as the deletion encompassed the splice acceptor consensus sequence of the exon, complementary RNA analysis was conducted to investigate its effect on splicing. The amplification of exons 7 to 10 of control cDNA led to the production of a 441 bp fragment. By contrast, the amplification of the patient’s cDNA identified an additional and predominant 248 bp fragment, supporting a splice defect (Figure 4A). Sanger sequencing of the RT-PCR products confirmed the presence of transcripts lacking the 193 bp of the *GSDME* exon 8 (r.991_1183del) in the patient (Figure 4B).

Familial segregation of the deletion was performed on available members of the family. All tested members with bilateral progressive NSHL (*n* = 10) were heterozygous carriers of the deletion c.991-60_1095del (Figure 1A). A 13-year-old boy (IV:5), who had normal hearing to date, was also a carrier. The deletion was not detected in three additional members who had normal audition (III:1, IV:1 and IV:3).

This variant was absent from all the interrogated databases. In accordance with the ACMG/AMP hearing loss guidelines, it was considered as a class V pathogenic variant.

### 3.2. Family S2106

**Figure 5:**
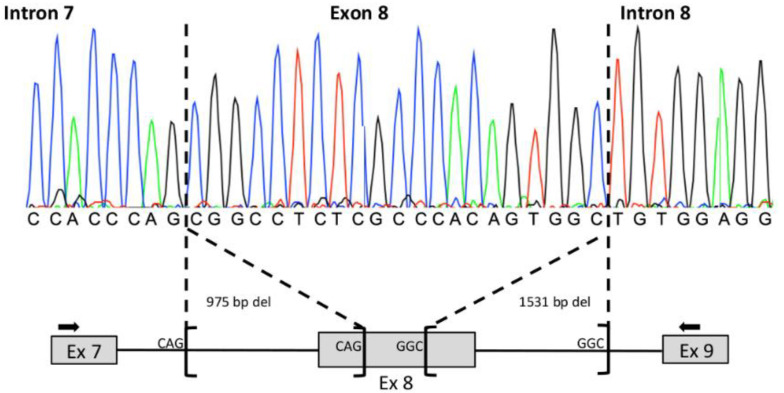
Sanger validation of the *GSDME* exon 8 deletion in the proband II:3. At the top: sequence chromatogram focused on the breakpoints of the deletions. At the bottom: schematic representation of the complex rearrangement. The position of the primers used for the amplification is shown by arrows. The 3 bp flanking microhomologies are indicated.

Molecular analysis of the proband II:3 by MPS identified the well-known class IV c.101T > C; p.(Met34Thr) *GJB2* variant and a single *GSDME* exon 8 deletion, both in the heterozygous state. Visual inspection of the reads, using the IGV tool, showed that the deletion breakpoints were not located in the 600 bp of the *GSDME* exon 8 target region. In order to validate this CNV and define its boundaries, a *GSDME* exon 7–exon 9 PCR was performed on the patient’s DNA. The PCR conditions employed in this study allowed a specific amplification of the mutated allele, and Sanger sequencing revealed a complex rearrangement (Figure 5).

This structural variant was composed of two deletions of 975 and 1531 bp, leading to the loss of the 17 first and 155 last bps of exon 8, respectively. Flanking microhomologies of 3 bp were detected for each deletion (Figure 5). According to the HGVS recommendations, this mutational event was described as c.[990+793_1007del; 1029_1183+1376del]. It was not reported in all the consulted databases and was classified as a class V pathogenic variant in accordance with the ACMG/AMP hearing loss guidelines.

## 4. Discussion and Conclusions

We described the molecular diagnostic investigations of two unrelated families suffering from hearing loss, which led to the identification of two pathogenic *GSDME* CNVs. Except for the proband II:3 of family S2106, who presented asymmetrical audiograms, all affected individuals exhibited a typical DFNA5 phenotype with post-lingual, bilateral, symmetric, predominantly high-frequency hearing loss. Asymmetrical hearing loss has previously been described in a DFNA5 patient [19], but there was no explanation for this atypical phenotype. Patient IV:5 of family S2426 was a 13-year-old boy carrying the familial *GSDME* deletion without any sign of hearing loss. As the onset of DFNA5-related hearing loss has been shown to occur between 0 and 50 years of life [5,13], an audiometric follow-up will be offered to this patient. In addition, as already described [11], intrafamilial variability in the age of onset can be noted. As an example, patient IV:5 is asymptomatic at the age of 13 years, whereas V:2 displayed HL in the high frequencies at the age of 9 years.

In the context of molecular genetic testing, gene panel sequencing using MPS is a powerful strategy to identify causal variants, including single-nucleotide variants, insertions, deletions or CNVs in patients referred for NSHL [20,21,22]. Custom computational tools have been successfully used to detect CNVs in hearing gene panels [15,21,23], but detection of true single-exon or partial exon deletions is still challenging. Due to a notable false positive rate, these single-exon CNVs are often not considered in routine MPS data analysis. Here, we identified two pathogenic single *GSDME* exon 8 CNVs, highlighting the crucial importance of carefully checking the read depth of this specific exon.

Deletions correspond to the second largest class of pathogenic variants recorded in the ClinVar database [24]. Furthermore, approximately 57% of them are flanked by microhomologies [25] that are hallmarks of the microhomology-mediated end joining (MMEJ) repair mechanism involved in re-ligation of DNA ends caused by double-strand breaks. In this study, the two described *GSDME* mutational events were also deletions flanked by microhomologies of 2 or 3 bp, demonstrating the implication of the MMEJ pathway.

As *GSDME* transcripts are expressed in whole blood, functional analysis was performed on the cDNA of proband IV:4 of the S2426 family in order to characterize the splice defect generated by the c.991-60_1095del variant. As expected, this deletion, in accordance with all previously described DFNA5-related pathogenic variations, led to exon 8 skipping and resulted in the translation of a truncated necrotic protein due to the loss of its GSDME-C domain.

In conclusion, we report here the two first single *GSDME* exon 8 CNVs implicated in DFNA5. These findings enrich the mutational spectrum of this gene and pinpoint the importance of accurate exploration of single-exon CNVs in a diagnostic service.

## Data Availability

All data are contained within the article. The variants, individuals and phenotypes described in this manuscript are available in the LOVD GVShared, Individual ID numbers #00396953 and #00396959.

## References

[1] Op de Beeck K., Van Camp G., Thys S., Cools N., Callebaut I., Vrijens K., Van Nassauw L., Van Tendeloo V.F.I., Timmermans J.P., Van Laer L. (2011). The DFNA5 Gene, Responsible for Hearing Loss and Involved in Cancer, Encodes a Novel Apoptosis-Inducing Protein. Eur. J. Hum. Genet..

[2] Rogers C., Fernandes-Alnemri T., Mayes L., Alnemri D., Cingolani G., Alnemri E.S. (2017). Cleavage of DFNA5 by Caspase-3 during Apoptosis Mediates Progression to Secondary Necrotic/Pyroptotic Cell Death. Nat. Commun..

[3] Zhang Z., Zhang Y., Xia S., Kong Q., Li S., Liu X., Junqueira C., Meza-Sosa K.F., Mok T.M.Y., Ansara J. (2020). Gasdermin E Suppresses Tumour Growth by Activating Anti-Tumour Immunity. Nature.

[4] De Schutter E., Croes L., Ibrahim J., Pauwels P., Op de Beeck K., Vandenabeele P., Van Camp G. (2021). GSDME and Its Role in Cancer: From behind the Scenes to the Front of the Stage. Int. J. Cancer.

[5] Bischoff A.M.L.C., Luijendijk M.W.J., Huygen P.L.M., van Duijnhoven G., De Leenheer E.M.R., Oudesluijs G.G., Van Laer L., Cremers F.P.M., Cremers C.W.R.J., Kremer H. (2004). A Novel Mutation Identified in the DFNA5 Gene in a Dutch Family: A Clinical and Genetic Evaluation. Audiol. Neurootol..

[6] Cheng J., Han D.Y., Dai P., Sun H.J., Tao R., Sun Q., Yan D., Qin W., Wang H.Y., Ouyang X.M. (2007). A Novel DFNA5 Mutation, IVS8+4 A>G, in the Splice Donor Site of Intron 8 Causes Late-Onset Non-Syndromic Hearing Loss in a Chinese Family. Clin. Genet..

[7] Chai Y., Chen D., Wang X., Wu H., Yang T. (2014). A Novel Splice Site Mutation in DFNA5 Causes Late-Onset Progressive Non-Syndromic Hearing Loss in a Chinese Family. Int. J. Pediatr. Otorhinolaryngol..

[8] Li-Yang M.-N., Shen X.-F., Wei Q.-J., Yao J., Lu Y.-J., Cao X., Xing G.-Q. (2015). IVS8+1 DelG, a Novel Splice Site Mutation Causing DFNA5 Deafness in a Chinese Family. Chin. Med. J. (Engl.).

[9] Chen S., Dong C., Wang Q., Zhong Z., Qi Y., Ke X., Liu Y. (2016). Targeted Next-Generation Sequencing Successfully Detects Causative Genes in Chinese Patients with Hereditary Hearing Loss. Genet. Test. Mol. Biomark..

[10] Booth K.T., Azaiez H., Kahrizi K., Wang D., Zhang Y., Frees K., Nishimura C., Najmabadi H., Smith R.J. (2018). Exonic Mutations and Exon Skipping: Lessons Learned from DFNA5. Hum. Mutat..

[11] Wang H., Guan J., Guan L., Yang J., Wu K., Lin Q., Xiong W., Lan L., Zhao C., Xie L. (2018). Further Evidence for “Gain-of-Function” Mechanism of DFNA5 Related Hearing Loss. Sci. Rep..

[12] Yuan Y., Li Q., Su Y., Lin Q., Gao X., Liu H., Huang S., Kang D., Todd N.W., Mattox D. (2020). Comprehensive Genetic Testing of Chinese SNHL Patients and Variants Interpretation Using ACMG Guidelines and Ethnically Matched Normal Controls. Eur. J. Hum. Genet..

[13] Yu C., Meng X., Zhang S., Zhao G., Hu L., Kong X. (2003). A 3-Nucleotide Deletion in the Polypyrimidine Tract of Intron 7 of the DFNA5 Gene Causes Nonsyndromic Hearing Impairment in a Chinese Family. Genomics.

[14] Van Laer L., Huizing E.H., Verstreken M., van Zuijlen D., Wauters J.G., Bossuyt P.J., Van de Heyning P., McGuirt W.T., Smith R.J., Willems P.J. (1998). Nonsyndromic Hearing Impairment Is Associated with a Mutation in DFNA5. Nat. Genet..

[15] Baux D., Vaché C., Blanchet C., Willems M., Baudoin C., Moclyn M., Faugère V., Touraine R., Isidor B., Dupin-Deguine D. (2017). Combined Genetic Approaches Yield a 48% Diagnostic Rate in a Large Cohort of French Hearing-Impaired Patients. Sci. Rep..

[16] Thorvaldsdóttir H., Robinson J.T., Mesirov J.P. (2013). Integrative Genomics Viewer (IGV): High-Performance Genomics Data Visualization and Exploration. Brief. Bioinform..

[17] den Dunnen J.T., Dalgleish R., Maglott D.R., Hart R.K., Greenblatt M.S., McGowan-Jordan J., Roux A.-F., Smith T., Antonarakis S.E., Taschner P.E.M. (2016). HGVS Recommendations for the Description of Sequence Variants: 2016 Update. Hum. Mutat..

[18] Oza A.M., DiStefano M.T., Hemphill S.E., Cushman B.J., Grant A.R., Siegert R.K., Shen J., Chapin A., Boczek N.J., Schimmenti L.A. (2018). Expert Specification of the ACMG/AMP Variant Interpretation Guidelines for Genetic Hearing Loss. Hum. Mutat..

[19] Park H.J., Cho H.J., Baek J.I., Ben-Yosef T., Kwon T.J., Griffith A.J., Kim U.-K. (2010). Evidence for a Founder Mutation Causing DFNA5 Hearing Loss in East Asians. J. Hum. Genet..

[20] Cabanillas R., Diñeiro M., Cifuentes G.A., Castillo D., Pruneda P.C., Álvarez R., Sánchez-Durán N., Capín R., Plasencia A., Viejo-Díaz M. (2018). Comprehensive Genomic Diagnosis of Non-Syndromic and Syndromic Hereditary Hearing Loss in Spanish Patients. BMC Med. Genom..

[21] Butz M., McDonald A., Lundquist P.A., Meyer M., Harrington S., Kester S., Stein M.I., Mistry N.A., Zimmerman Zuckerman E., Niu Z. (2020). Development and Validation of a Next-Generation Sequencing Panel for Syndromic and Nonsyndromic Hearing Loss. J. Appl. Lab. Med..

[22] Abu Rayyan A., Kamal L., Casadei S., Brownstein Z., Zahdeh F., Shahin H., Canavati C., Dweik D., Jaraysa T., Rabie G. (2020). Genomic Analysis of Inherited Hearing Loss in the Palestinian Population. Proc. Natl. Acad. Sci. USA.

[23] Tropitzsch A., Schade-Mann T., Gamerdinger P., Dofek S., Schulte B., Schulze M., Battke F., Fehr S., Biskup S., Heyd A. (2021). Diagnostic Yield of Targeted Hearing Loss Gene Panel Sequencing in a Large German Cohort With a Balanced Age Distribution from a Single Diagnostic Center: An Eight-Year Study. Ear Hear..

[24] Rees H.A., Liu D.R. (2018). Base Editing: Precision Chemistry on the Genome and Transcriptome of Living Cells. Nat. Rev. Genet..

[25] Grajcarek J., Monlong J., Nishinaka-Arai Y., Nakamura M., Nagai M., Matsuo S., Lougheed D., Sakurai H., Saito M.K., Bourque G. (2019). Genome-Wide Microhomologies Enable Precise Template-Free Editing of Biologically Relevant Deletion Mutations. Nat. Commun..

